# Influence of Body Composition Assessed by Computed Tomography on Mortality Risk in Young Women with Breast Cancer

**DOI:** 10.3390/nu16183175

**Published:** 2024-09-19

**Authors:** Agnes Denise de Lima Bezerra, Jarson Pedro da Costa Pereira, Ingryd Fernandes de Macedo Soares, Glaucia Mardrini Cassiano Ferreira, Ana Lúcia Miranda, Galtieri Otávio Cunha de Medeiros, Sara Maria Moreira Lima Verde, Ana Paula Trussardi Fayh

**Affiliations:** 1 Postgraduate Program in Health Sciences, Health Sciences Centre, Federal University of Rio Grande do Norte, Natal 59078-970, Brazil; agnes_denise@hotmail.com (A.D.d.L.B.); glauciamardrine@yahoo.com.br (G.M.C.F.); analucia_nutri@yahoo.com.br (A.L.M.); galtieri_otavio@yahoo.com.br (G.O.C.d.M.); 2 Postgraduate Program in Nutrition, Department of Nutrition, Federal University of Pernambuco, Recife 50670-901, Brazil; jarson.costa@ufpe.br; 3 Postgraduate Program in Nutrition and Health, State University of Ceara, Fortaleza 60714-903, Brazil; ingrydfernandes@hotmail.com (I.F.d.M.S.); sara.maria@uece.br (S.M.M.L.V.); 4 PesqClin Lab, Onofre Lopes University Hospital, Brazilian Company of Hospital Services (EBSERH), Federal University of Rio Grande do Norte, Natal 59078-970, Brazil

**Keywords:** body composition, breast cancer, fat mass, muscle mass, prognosis, mortality

## Abstract

**Background**/**Objectives:** Increasing evidence indicates that body composition can significantly influence prognosis in women with breast cancer. However, alterations in body composition, particularly among young women (<40 years), remain largely unknown and underexplored. This study aimed to investigate the relationship of computed tomography (CT)-derived body composition with mortality rates among young women recently diagnosed with breast cancer, identifying the best-correlated cutoff value. **Methods:** This is a bi-set cohort study with retrospective data collection. Women newly diagnosed with ductal invasive breast cancer, aged 20 to 40 years, treated in reference oncology units were included. Body composition was assessed using CT scans at the third lumbar vertebra (L3) level, including muscle and adipose compartments. The outcome of interest was the incidence of overall mortality. A maximally selected log-rank Cox-derived analysis was employed to assess the cutoffs associated with mortality. **Results:** A total of 192 women were included before any form of treatment (median age of 35 years, IQ range: 31–37). Overall mortality occurred in 12% of the females. Stages III–IV were the most frequent (69.5%). Patients who died had a significantly lower muscle area index. CT-derived muscle area was inversely associated with mortality. Each 1 cm^2^/m^2^ decrease in skeletal muscle index increased the mortality hazard by 9%. Higher values of adiposity compartments were independently associated with higher mortality. **Conclusions:** Our study highlights the predictive significance of skeletal muscle area and adipose tissue in predicting survival among young women recently diagnosed with breast cancer.

## 1. Introduction

Breast cancer is the most prevalent type of cancer worldwide, significantly affecting females in Brazil [1,2]. Breast cancer contributes to mortality rates, with a risk equivalent to 16.5 deaths per 100 thousand women [1,2,3]. The incidence of breast cancer is rising due to several factors, including demographic aging, overdiagnosis consequent to excessive screening, and lifestyle factors, such as excess weight and its features [4,5]. Evidence indicates that excess weight or weight gain following diagnosis correlates with elevated risks of breast cancer mortality, all-cause mortality, and cancer recurrence [6,7,8].

Although evidence often connects aging and excess weight to both breast cancer development and specific mortality [8,9], a troubling trend has emerged: the rising incidence of breast cancer among young women [10,11,12]. In contrast, there is a paradoxical debate suggesting that excess weight (e.g., overweight or obesity) may be associated with a more favorable prognosis in this clinical population [13,14]. These results are controversial because the impact of obesity may vary depending on the stage of breast cancer disease (early vs. advanced). One study found that obesity was associated with improved overall survival in advanced breast cancer, yet correlated with poorer overall survival in early-stage disease [14]. However, most studies use body mass index (BMI) to diagnose obesity [14]. While BMI is a widely used indicator of adiposity, it does not differentiate between fat and skeletal muscle (SM) or account for body fat distribution [15]. BMI may overlook critical aspects of body composition, such as muscle depletion or excess visceral fat, both of which may significantly impact cancer outcomes [15]. It is also recognized that individuals with excess weight (higher BMI) may develop greater SM mass to support the added weight [15]. This increased SM could partially explain the paradoxical effect observed in some studies [15].

In this context, the relationship of body composition with breast cancer survival remains to be explored [16,17]. This is particularly relevant among young women diagnosed with breast cancer, who are often assumed to be at their peak body composition [18]. While body composition abnormalities are more commonly associated with aging, they can also occur at younger ages [18]. Although much of the research has focused on older populations, growing evidence suggests that these abnormalities may predict poor outcomes regardless of age [19]. This is critical, given the significant metabolic roles that both muscle and fat compartments play, potentially affecting treatment response and overall prognosis [20].

Given that young women with the same BMI can exhibit significant differences in body composition [21], such an evaluation becomes particularly relevant. In oncology, patients can benefit from opportunistic body composition assessment through computed tomography (CT), which provides precise measurements of various body compartments, including SM quantity, muscle composition, and adipose tissue distribution [22,23,24]. This method allows for a more comprehensive understanding of how body composition impacts cancer prognosis, beyond the limitations of BMI alone. As mentioned before, muscle and adipose tissues may be relevant predictors of prognosis in cancer, affecting clinical outcomes such as chemotoxicity and mortality [25,26].

Although previous studies have included a wide age range and demonstrated that body composition abnormalities can affect clinical outcomes in cancer regardless of age, there remains a significant gap in studies focusing on younger patients newly diagnosed with breast cancer. This lack of targeted studies limits our understanding of how body composition influences prognosis in this population, highlighting the need for further investigation in this area. We hypothesized that body composition abnormalities could predict poor survival, even in younger populations with breast cancer. If our hypothesis is confirmed, this detection could potentially facilitate the development of timely and tailored interventions aimed at enhancing prognosis. Thus, our study aimed to investigate the relationship between CT-derived body composition parameters and mortality rates among young women recently diagnosed with breast cancer, in addition to identifying the best cutoff values that predict this outcome.

## 2. Methods

### 2.1. Study Design and Subjects

**Figure 1:**
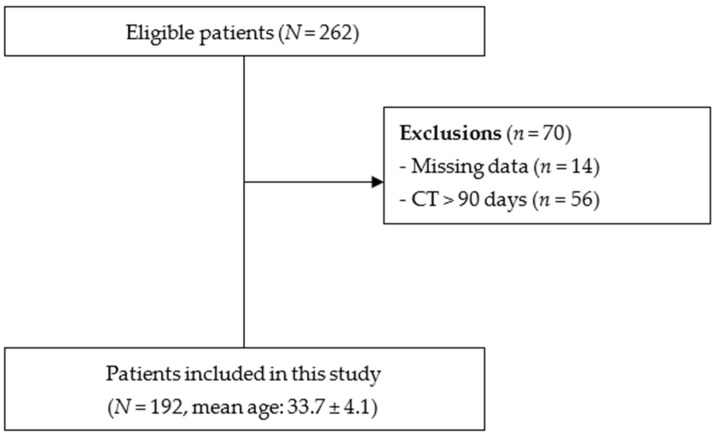
Study flowchart.

This is a cohort study with retrospective data collection. A cohort of young females, aged between 20–40 years, newly diagnosed with invasive ductal breast cancer (stages I to IV) were conveniently sampled. They were under outpatient care in two reference oncology centers in Northeast Brazil. An additional inclusion criterion was the availability of abdominal CT scans for diagnosis or staging, without a previous history of cancer. Data collection occurred between January 2022 and July 2023. Patients with clinical or anatomical conditions that hindered CT evaluations were excluded from this analysis (e.g., ascites or anasarca). Those with missing anthropometric data in electronic medical records, CT scans > 90 days from the date of diagnosis, and patients who already underwent any type of treatment were also excluded. This study initially screened 262 women potentially eligible. A total of 70 participants were excluded due to missing data, and for presenting CT > 90 days from the date of diagnosis. A flowchart of patients’ selection is presented in Figure 1. This study was approved by the institutions’ Research Ethics Committees (CAAE: 43488621.4.0000.5528). Informed consent was waived. 

### 2.2. Outcomes and Covariates

Sociodemographic, clinical, and anthropometric information was obtained from the electronic medical records. Data on age, staging, histological subtype, hormonal receptors, and long-term mortality were registered for analysis. On anthropometry, body weight (kg) and height (m) were registered to calculate BMI (kg/m^2^), classified according to the World Health Organization [27]. Anthropometric data were measured by the nutrition team at the respective units.

### 2.3. Body Composition from Computed Tomography Scans

Body composition was assessed at the third lumbar vertebra (L3) level, which is commonly used in oncology studies due to its strong correlation with whole-body composition and its reliability in evaluating muscle and adipose tissue [22]. L3 images were analyzed using the Slice-O-Matic software (v.4.3, Tomovision^®^, Montreal, QC, Canada), which allows specific tissue demarcation to be specified using Hounsfield unit thresholds: from −29 to +150 for SM (psoas, erector spinal, quadratus lumborum, transversus abdominis, internal and external obliques, rectus abdominis), from −150 to −50 for visceral adipose tissue (VAT), and from −190 to −30 for subcutaneous and intramuscular adipose tissue (SAT and IMAT, respectively). Total adipose tissue (TAT) was determined by the sum of the VAT, SAT, and IMAT measures. The skeletal muscle index (SMI) was determined by the SMA value, corrected by height in m^2^. All CT images were analyzed by a single trained researcher with anatomical expertise (G. O. C. M.). The intraclass correlation coefficient (ICC) and its 95% confidence interval (CI) were assessed using the same 15 images, analyzed over six distinct months. This evaluation was based on a single measurement, employing the absolute agreement, 2-way mixed-effects model. The ICC and CI for repeated measurements were 1.0 (95% CI: 1.0–1.0).

### 2.4. Statistical Analyses

Data were analyzed using the R studio version 4.3.2, and the Statistical Package for the Social Sciences (SPSS), version 20.0 (SPSS Inc., Chicago, IL, USA). Data normality was assessed using the Shapiro–Wilk test. Normally distributed data were described using mean ± standard deviation (SD), compared through independent Student’s *t*-tests. Non-normally distributed data were described using medians and interquartile (IQ) ranges, compared through the Mann–Whitney U test. Categorical variables were described as absolute (*N*) and relative (%) frequencies, compared using Pearson’s χ^2^ test or Fisher’s exact test. Adhering to Schoenfeld residual assumptions, Cox proportional hazards analysis was conducted to investigate the relationship between body composition and mortality. The body composition variables that independently correlated with mortality (adjusted hazards) underwent maximally selected log-rank analysis to determine optimal cutoff points. Adjustments were based on the prognostic criterion (TNM stage) and variations in BMI, to minimize the influence of body size variations. To estimate the mortality risk associated with a decrease in body composition parameters (continuous variable), the reciprocal of the hazard ratio (1/HR) was calculated. Statistical significance was established at *p* < 0.05 for all analyses.

Although it is a debatable approach, a post-hoc sample power analysis was also conducted using R Studio version 4.3.2. This analysis was based on the significant differences in SMI between survivors (47.1 ± 7.4 cm^2^/m^2^) and non-survivors (42.5 ± 8.0 cm^2^/m^2^), with sample sizes of 169 and 23, respectively. Effect size was calculated using Cohen’s method, and the test was performed with a 5% significance level and a two-tailed approach. The results indicated a power (1 − β) of 76.2%.

## 3. Results

**Figure 2:**
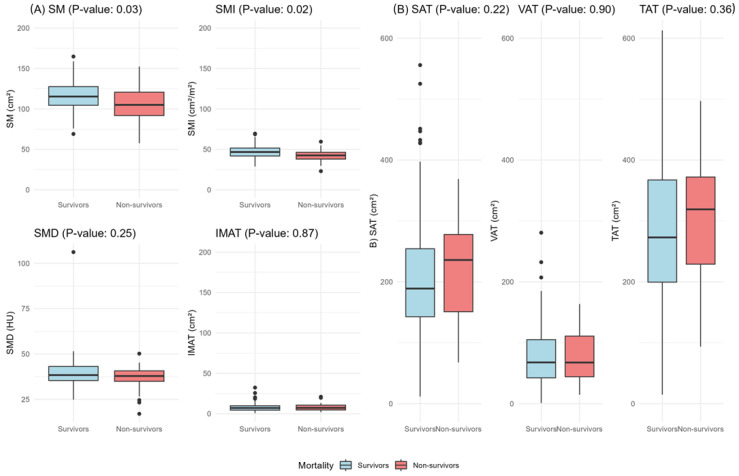
Comparative analysis of CT-based body composition between young women (20–40 years) with breast cancer who survived (blue plots) and those who did not survive (red plots). (**A**) Means muscle compartments: SM, SMI, SMD, IMAT. (**B**) Means fat compartments: SAT, VAT, TAT. Mann–Whitney U test for medians, independent Student’s *t*-test for means. Abbreviations: SM: skeletal muscle; SMI: skeletal muscle index in cm^2^/m^2^; SMD: skeletal muscle radiodensity in Hounsfield Units (HU); IMAT: intramuscular adipose tissue; SAT: subcutaneous adipose tissue; VAT: visceral adipose tissue; TAT: total adipose tissue.

A total of 192 young women were included in this analysis (Figure 1). Most of them identified as non-Caucasian (85.4%) and lived with a partner (61.9%). The median age at the time of diagnosis was 35 years (IQ range: 31–37). Up to 6 years of follow-up, the mortality incidence occurred in 12% (*n* = 23). A detailed overview of the sociodemographic and clinical features of the patients is provided in Table 1. Table 2 presents the nutritional assessment of patients, complemented by visual representation in Figure 2. The patients who died presented with both lower SM (cm^2^) (*p* = 0.009) and SMI (cm^2^/m^2^) (*p* = 0.015), with no differences for the other body composition compartments.

**Table 1:** Characteristics of young females newly diagnosed with breast cancer (*N* = 192).

Variables	Total	Survivors	Non-Survivors	*p*
*N*, %	192	169 (88.0)	23 (12.0)	
Ethnicity, *n* (%)				0.09
Caucasian	28 (14.6)	22 (78.6)	6 (21.4)	
Non-Caucasian	164 (85.4)	147 (89.6)	17 (10.4)	
Educational level, *n* (%)				0.84
Elementary	66 (35.5)	57 (86.4)	9 (13.6)	
Secondary	85 (45.7)	76 (89.4)	9 (10.6)	
Post-secondary	35 (18.8)	31 (88.6)	4 (11.4)	
Marital status, *n* (%)				0.51
With partner	117 (61.9)	102 (87.2)	15 (12.8)	
No partner	72 (38.1)	65 (90.3)	7 (9.7)	
Age at diagnosis, y (median IQR)	35 (31–37)	35 (31–37)	34 (31–37)	0.63
Menarche, y (median IQR)	13 (12–14)	13 (12–14)	13 (11.5–14.5)	0.89
Presence of comorbidities, *n* (%)				
Type 2 diabetes mellitus	3 (1.6)	3 (100.0)	0 (0.00)	0.52
Hypertension	21 (89.1)	18 (85.7)	3 (14.3)	0.73
Dyslipidemia	3 (1.6)	3 (100.0)	0 (0.00)	0.52
TNM stage, *n* (%)				**0.014**
I and II	58 (30.5)	56 (96.6)	2 (3.4)	
III and IV	132 (69.5)	112 (84.8)	20 (15.2)	
Lymph node status at diagnosis, *n* (%)				**0.046**
Positive	153 (79.9)	129 (85.4)	22 (14.6)	
Negative	36 (19.0)	36 (100)	0 (0.0)	
Unknown	2 (1.1)	1 (50.0)	1 (50.0)	
Estrogen receptor status, *n* (%)				0.30
Positive	112 (60.5)	102 (91.1)	10 (8.9)	
Negative	73 (39.5)	63 (86.3)	10 (13.7)	
Progesterone receptor status, *n* (%)				0.20
Positive	107 (58.2)	98 (91.6)	9 (8.4)	
Negative	77 (41.8)	66 (85.7)	11 (14.3)	
HER2 status, *n* (%)				0.64
Positive	61 (33.0)	55 (90.2)	6 (9.8)	
Negative	124 (67.0)	109 (87.9)	15 (12.1)	
Immunophenotype, *n* (%)				0.77
Luminal A	14 (8.9)	13 (12.9)	1 (7.1)	
Luminal B	54 (34.2)	48 (88.9)	6 (11.1)	
Overexpression of ER2-HER2+	15 (9.5)	42 (89.4)	5 (10.6)	
Triple-negative	47 (29.7)	14 (93.3)	1 (6.7)	
Unknown	28 (17.7)	23 (82.1)	5 (17.9)	
Ki67 status, *n* (%)				0.75
≤20	45 (28.5)	39 (86.7)	6 (13.3)	
>20	113 (71.5)	100 (88.5)	13 (11.5)	

Data in frequency (*n*, %—compared using χ^2^ test or Fisher’s exact test) or median and IQR: interquartile range (compared using Mann–Whitney U test). Notes and sample size: educational level (*n* = 186); estrogen receptor status (*n* = 185); HER2: human epidermal growth factor receptor 2 (*n* = 185); immunophenotype (*n* = 158); ki67 status (*n* = 158); lymph nodes (*n* = 189); marital status (*n* = 189); menarche (*n* = 181); progesterone receptor status (*n* = 184). Bold is used to emphasize significant *p*-values.

**Table 2:** Anthropometric and body composition assessment of young females newly diagnosed with breast cancer (*N* = 192).

Variables	Total	Survivors	Non-Survivors	*p*
BMI (kg/m^2^), (median IQR)	26.2 (23.5–29.8)	26.2 (23.5–29.7)	27.0 (21.7–30.1)	0.80 ^a^
Underweight, *n* (%)	3 (1.6)	3 (1.8)	0 (0.0)	0.77 ^b^
Normal range, *n* (%)	71 (37.0)	61 (36.1)	10 (43.5)	
Overweight, *n* (%)	72 (37.5)	65 (38.5)	7 (30.4)	
Obesity, *n* (%)	46 (24.0)	40 (23.7)	6 (26.1)	
CT-derived body composition, (median IQR)				
SAT (cm^2^)	190.3 (142.7–259.3)	189.0 (142.5–254.6)	235.9 (149.3–283.8)	0.22 ^a^
VAT (cm^2^)	67.5 (42.4–107.5)	67.6 (39.3–106.7)	67.5 (43.6–116.9)	0.90 ^a^
TAT (cm^2^)	276.3 (197.7–367.5)	272.9 (198.3–367.4)	319.0 (183.8–384.0)	0.36 ^a^
IMAT (cm^2^)	7.25 (4.57–10.01)	7.24 (4.57–9.99)	7.36 (4.44–11.05)	0.87 ^a^
SMD (HU)	38.2 (35.3–43.1)	38.4 (35.4–43.1)	37.8 (34.6–41.2)	0.25 ^a^
SM (cm^2^)	114.3 ± 18.8	115.6 ± 18.1	104.8 ± 21.1	**0.029 ^c^**
SMI (cm^2^/m^2^)	46.5 ± 7.6	47.1 ± 7.4	42.5 ± 8.0	**0.015 ^c^**

^a^ Mann–Whitney U test (median and IQR: interquartile range); ^b^ Pearson’s χ^2^ test (frequencies, *N* %); ^c^ independent Student’s *t*-test (mean ± standard deviation). Abbreviations: BMI: body mass index; CT: computed tomography; SAT: subcutaneous adipose tissue; VAT: visceral adipose tissue; TAT: total adipose tissue; IMAT: intramuscular adipose tissue; SMD: skeletal muscle radiodensity in Hounsfield Units (HU); SM: skeletal muscle; SMI: skeletal muscle index in cm^2^/m^2^. Bold is used to emphasize significant *p*-values.

Cox regression analysis using continuous variables demonstrated that only SM, SMI, SAT, and TAT were independently associated with mortality. Each decrease in one cm^2^ and cm^2^/m^2^ of SM and SMI, respectively, were associated with a 3–9% increase in the hazard of mortality. In addition, higher values of both SAT and TAT were independently associated with a high mortality rate. A statistical tendency towards SMD was observed, and it also underwent maximally selected log-rank cutoffs. Table 3 illustrates detailed cutoffs and hazard coefficients for mortality.

## 4. Discussion

This study is the first to explore associations between CT-based body composition and mortality in a cohort of young women newly diagnosed with breast cancer in Latin America, providing survival-specific cutoff values related to body composition. Our results show that increases in both SM and SMI independently decrease the hazard of mortality, while increases in SAT and TAT are associated with a higher mortality risk. These preliminary findings emphasize the prognostic importance of body composition in predicting survival outcomes in young women with breast cancer.

Our findings are consistent with previous studies showing that low SM and excess adiposity are linked to higher mortality in breast cancer patients, regardless of the TNM stage [25]. While this relationship is well-established in older populations, our study highlights that similar associations hold true for younger women. Although our hazard estimates for mortality associated with adiposity were lower, this may be due to the relatively small sample size. Nevertheless, even small risks could have clinical relevance, reinforcing the importance of addressing body composition abnormalities.

Based on our body composition results, our study reinforces the inadequacy of relying solely on BMI for nutritional assessment, as it overlooks subtle or critical variations in body composition. Although only three patients were classified as underweight and none died, more than 60% were categorized as overweight or with obesity. Despite the ‘obesity paradox’ reported in similar populations [13,28], our findings show that low SM and higher adiposity are independent risk factors for mortality, even among those with higher body mass. Therefore, the early detection of body composition abnormalities could significantly improve survival outcomes and nutritional status.

Our additional results identified new mortality-related cutoffs for SM (<94.2 cm^2^) and SMI (<39.0 cm^2^/m^2^), closely aligning with previously established thresholds in patients with breast cancer [26,29]. However, our study exclusively focuses on young women aged 20–40, a population underrepresented in previous research. The validation of cutoff values specific to this demographic is important, as social, epidemiological, and biological factors can vary by age. The consistency of our results with prior studies supports potential generalizability to similar populations. In addition to SMI, we evaluated SM in absolute terms (cm^2^), which is rarely reported without height adjustment. This approach introduces a novel dimension to assessing low SM concerning mortality. However, SM alone may not fully account for individual anatomical variations, and further studies are needed to refine this method.

Few studies have examined the relationship between body composition and survival in patients with breast cancer, most of them focusing on metastatic disease or mixed-age populations [25,29,30,31,32,33]. For instance, a large study of 3283 non-metastatic breast cancer patients reported that 34% had low SMI (<40 cm^2^/m^2^), which was associated with higher mortality [30]. Similarly, a study of 3241 women found that low SM correlated with increased mortality [29]. These findings align with our results, where each 1 cm^2^/m^2^ decrease in SMI increased the mortality hazard by 9%.

This study also reinforces the theoretical aspect of SM as a functional, endocrine, and immunological organ [34,35,36]. The precise mechanisms linking muscle to survival following a cancer diagnosis remain unclear, but they may occur through various physiological and metabolic pathways. This includes inflammation and behavioral pathways, including reduced physical activity due to deconditioning and fatigue [26,37]. Muscle has local autocrine, paracrine, and endocrine effects, but also secretes cytokines and other myokines (including IL-6, IL-8, IL-15, and leukemia inhibitory factor), leading to systemic effects [36,38]. Therefore, higher amounts of muscle may diminish the impact of inflammation, while lower levels of muscle can lead to local or aggravate systemic inflammation [39], potentially affecting outcomes.

Systemic inflammation can also contribute to persistent muscle wasting in patients with cancer [40,41]. Consequently, a dual pathway to mortality may ensue: cancer-induced muscle wasting may occur, alongside muscle wasting exacerbating clinical conditions in cancer [26,40]. Muscle loss can also disrupt the oxidative pathways, stimulating tumor growth [42], and is associated with insulin resistance [43]. Low SM not only affects survival post-cancer diagnosis but also impacts critical factors, such as surgical complications, treatment toxicity, and the potential need for chemotherapy dose reductions or interruptions [44,45]. These factors can compromise the effectiveness of adjuvant therapies administered with curative intent [26,46]. Moreover, these substantial muscle abnormalities may extend, potentially leading to increased functional dependence, frailty, reduced autonomy, and a decline in overall quality of life [47,48].

This study is not without limitations. The retrospective data collection cohort design introduces the potential for selection bias. This may have occurred due to the exclusion of patients with incomplete data or those whose CT scans were performed more than 90 days after diagnosis. Such exclusions could result in a sample that is not fully representative of the broader breast cancer population, particularly younger patients or those with more advanced disease, who might have experienced delays in imaging. However, these time points were chosen to minimize the impact of changes in body composition over time. Additionally, this study’s retrospective design means that data collection was not standardized, which may introduce further variability and bias. The exclusive reliance on clinical records for data collection also introduces the potential for information bias and missing or incomplete data, such as detailed information on treatment procedures and comprehensive clinical characteristics.

The relatively small sample size, with limited statistical power (76%), although close to the commonly accepted threshold of 80%, may limit the generalizability of our findings. This limitation persists despite this study being conducted in two large cancer treatment centers, highlighting the need for cautious interpretation when applying these results to broader populations. This caution is particularly important, as our results may not fully represent the broader breast cancer Brazilian population, which can vary across different healthcare settings (primary, secondary, tertiary care), private and public hospitals, as well as socioeconomic factors. These variations could influence the outcomes and limit the generalizability of our findings to other patient populations. Furthermore, some unmeasured factors could have impacted the results in the regression model. Despite these challenges, the small sample size may be additionally influenced by the relatively lower incidence of breast cancer in younger ages, which, in turn, strengthens the relevance of our findings. As a result, our study provides valuable insights into a population that is often underrepresented in breast cancer research.

Additional positive aspects of our study include a relatively homogeneous population, consisting of young women recently diagnosed with both metastatic and non-metastatic breast cancer. These patients exhibited no significant clinical differences in the initiation of systemic treatment. Furthermore, our cohort was followed up for 5 years across two large reference centers for oncological treatment, in addition to the utilization of CT scans—for detailing body composition, reinforcing the precision and accuracy of our findings. As future implications and applicability, we recommend that clinicians (dietitians, oncologists) should, whenever feasible, assess CT scans to evaluate body composition of these patients. This approach may assist in diagnosing abnormal nutritional statuses (i.e., sarcopenia, sarcopenic obesity, and malnutrition), and aid in prognostication. However, we also acknowledge that this depends on the opportunistic availability of CT images and the training required to use specialized software for body composition analysis.

Despite the potential of our findings, future prospective studies with larger sample sizes and comprehensive prospective data collection methods are necessary to further elucidate the relationship between body composition and mortality in young patients with breast cancer. These studies could further explore the relationship between body composition and clinical outcomes by accounting for differences in ethnicity, as these factors may vary, particularly concerning tumor hormone receptor subtype distribution [49]. Such differences could potentially influence the results, highlighting the importance of considering ethnic variations in future research to better understand their impact on cancer outcomes. When CT scans are not available, studies could benefit from investigating BMI and other anthropometric markers of body composition, such as waist circumference and BMI-adjusted calf circumference [50,51]. These measures may provide useful estimates of obesity and low SM, offering an alternative approach to body composition-related prognosis.

## 5. Conclusions

This study demonstrates that abnormalities in body composition predict survival in a population of young women newly diagnosed with breast cancer. Our findings particularly highlight the importance of skeletal muscle and adiposity as predictors of precocious death in this cohort. Despite the need for further studies to validate our findings, our results are relevant for patient care. Clinicians and researchers working with similar populations can use our identified cutoffs for comparison and to assist in nutritional diagnosis. This is particularly important, given that body composition is a modifiable risk factor for mortality, highlighting the critical role of early detection and timely intervention to improve patient outcomes.

## Figures and Tables

**Table 3:** Cox regression analysis: Associations between body composition and mortality of young females newly diagnosed with breast cancer (*N* = 192).

Variables	HR_crude (95% CI)_	*p*	HR_adjusted (95% CI)_	*p*	Cutoffs
SM (cm^2^)	0.97 (0.95;0.99)	**0.026**	0.97 (0.94;0.99)	**0.014**	<94.2
SMI (cm^2^/m^2^)	0.93 (0.88;0.99)	**0.018**	0.91 (0.85;0.97)	**0.004**	<39
SMD (HU)	0.93 (0.88;0.99)	**0.029**	0.94 (0.88;1.01)	0.07	<42.5
IMAT (cm^2^)	1.03 (0.96;1.11)	0.46	1.01 (0.92;1.10)	0.90	
SAT (cm^2^)	1.00 (0.99;1.01)	0.29	1.01 (1.00;1.02)	**0.030**	>216
VAT (cm^2^)	1.00 (0.99;1.01)	0.37	1.00 (0.99;1.02)	0.26	
TAT (cm^2^)	1.00 (0.99;1.00)	0.27	1.01 (1.00;1.01)	**0.021**	>273

Adjusted models mean that all body composition variables were adjusted for TNM stage and body mass index. Abbreviations: SM: skeletal muscle; SMI: skeletal muscle index in cm^2^/m^2^; SMD: skeletal muscle radiodensity in Hounsfield Units (HU); IMAT: intramuscular adipose tissue; SAT: subcutaneous adipose tissue; VAT: visceral adipose tissue; TAT: total adipose tissue. Bold is used to emphasize significant *p*-values.

## Data Availability

The original contributions presented in the study are included in the article. Further inquiries can be directed to the corresponding author.
